# Effect of rituximab on a salivary gland ultrasound score in primary Sjögren’s syndrome: results of the TRACTISS randomised double-blind multicentre substudy

**DOI:** 10.1136/annrheumdis-2017-212268

**Published:** 2017-12-23

**Authors:** Benjamin A Fisher, Colin C Everett, John Rout, John L O’Dwyer, Paul Emery, Costantino Pitzalis, Wan-Fai Ng, Andrew Carr, Colin T Pease, Elizabeth J Price, Nurhan Sutcliffe, Jimmy Makdissi, Anwar R Tappuni, Nagui S T Gendi, Frances C Hall, Sharon P Ruddock, Catherine Fernandez, Claire T Hulme, Kevin A Davies, Christopher John Edwards, Peter C Lanyon, Robert J Moots, Euthalia Roussou, Andrea Richards, Linda D Sharples, Michele Bombardieri, Simon J Bowman

**Affiliations:** 1 National Institute for Health Research (NIHR), Birmingham Biomedical Research Centre, Birmingham, UK; 2 Rheumatology Research Group, Institute of Inflammation and Ageing, University of Birmingham, Birmingham, UK; 3 Rheumatology Department, University Hospitals Birmingham NHS Foundation Trust, Birmingham, UK; 4 Leeds Institute for Clinical Trials Research, University of Leeds, Leeds, UK; 5 Birmingham Dental Hospital, Birmingham, UK; 6 Academic Unit of Health Economics, Leeds Institute of Health Sciences, University of Leeds, Leeds, UK; 7 Biomedical Research Centre, Leeds Teaching Hospitals NHS Trust, Leeds, UK; 8 Leeds Institute of Rheumatic and Musculoskeletal Medicine, University of Leeds, Chapel Allerton Hospital, Leeds, UK; 9 William Harvey Research Institute, Barts and The London School of Medicine and Dentistry, London, UK; 10 Institute of Cellular Medicine, University of Newcastle, Newcastle-upon-Tyne, UK; 11 Newcastle Dental Hospital, Newcastle-upon-Tyne, UK; 12 Great Western Hospital, Swindon, UK; 13 Royal London Hospital, Barts Health NHS Trust, London, UK; 14 Institute of Dentistry, Barts and The London School of Medicine and Dentistry, Queen Mary University of London, London, UK; 15 Basildon and Thurrock University Hospital, Basildon, UK; 16 Department of Clinical Medicine, University of Cambridge, Addenbrookes Hospital, Cambridge, UK; 17 Brighton and Sussex Medical School, University of Sussex, Brighton, UK; 18 NIHR Wellcome Trust Clinical Research Facility, University Hospital Southampton, Southampton, UK; 19 Nottingham University Hospitals NHS Trust, and Nottingham NHS Treatment Centre, Nottingham, UK; 20 Department of Musculoskeletal Biology, Institute of Ageing and Chronic Disease, University of Liverpool, Liverpool, UK; 21 Barking Havering and Redbridge University Hospitals NHS trust (BHRUT), King George Hospital, Goodmayes, UK; 22 Department of Medical Statistics, London School of Hygiene and Tropical Medicine, London, UK

**Keywords:** sjøgren’s syndrome, ultrasonography, B cells

## Abstract

**Objectives:**

To compare the effects of rituximab versus placebo on salivary gland ultrasound (SGUS) in primary Sjögren’s syndrome (PSS) in a multicentre, multiobserver phase III trial substudy.

**Methods:**

Subjects consenting to SGUS were randomised to rituximab or placebo given at weeks 0, 2, 24 and 26, and scanned at baseline and weeks 16 and 48. Sonographers completed a 0–11 total ultrasound score (TUS) comprising domains of echogenicity, homogeneity, glandular definition, glands involved and hypoechoic foci size. Baseline-adjusted TUS values were analysed over time, modelling change from baseline at each time point. For each TUS domain, we fitted a repeated-measures logistic regression model to model the odds of a response in the rituximab arm (≥1-point improvement) as a function of the baseline score, age category, disease duration and time point.

**Results:**

52 patients (n=26 rituximab and n=26 placebo) from nine centres completed baseline and one or more follow-up visits. Estimated between-group differences (rituximab-placebo) in baseline-adjusted TUS were −1.2 (95% CI −2.1 to −0.3; P=0.0099) and −1.2 (95% CI −2.0 to −0.5; P=0.0023) at weeks 16 and 48. Glandular definition improved in the rituximab arm with an OR of 6.8 (95% CI 1.1 to 43.0; P=0.043) at week 16 and 10.3 (95% CI 1.0 to 105.9; P=0.050) at week 48.

**Conclusions:**

We demonstrated statistically significant improvement in TUS after rituximab compared with placebo. This encourages further research into both B cell depletion therapies in PSS and SGUS as an imaging biomarker.

**Trial registration number:**

65360827, 2010-021430-64; Results.

## Introduction

Primary Sjögren’s syndrome (PSS) is characterised by focal lymphocytic infiltration of exocrine glands leading to profound dryness. It is often accompanied by systemic manifestations and high levels of fatigue. B cells are considered to have a central role in pathogenesis,^1^ and two small randomised controlled trials (RCTs) of the anti-CD20 B-cell-depleting agent rituximab suggested benefits in PSS.^2 3^ Rituximab may also have effects on interleukin-17-producing mast cells and on a CD20-positive T cell subset.^4 5^ Despite this, French (TEARS) and British (TRACTISS) phase III RCTs failed to demonstrate an effect on primary endpoints based on patient-reported visual analogue scales (VAS).^6 7^ Potential explanations for these disappointing findings include the lack of patient stratification, insufficient tissue depletion of B cells and the choice and timing of primary outcome.

The requirement for new and validated outcome measures for PSS led to the development of the European Sjögren’s Syndrome Patient Reported Index (ESSPRI) and a physician-assessed systemic disease activity index (European League Against Rheumatism Sjögren’s Syndrome Disease Activity Index (ESSDAI)).^8^ These are a welcome advance, but certain limitations suggest that additional objective outcome measures/biomarkers would be desirable. Use of the ESSDAI, for example, requires a minimum threshold for trial entry that excludes a large proportion of patients. Other outcome measures include salivary flow rates, although these are subject to issues of standardisation and diurnal variation,^9^ and histological examination of salivary gland biopsies, which may provide mechanistic information but is invasive.^10 11^ Salivary gland ultrasound (SGUS) is readily available, non-invasive and shows reasonable sensitivity and good specificity for the diagnosis of PSS.^12–14^ In PSS, glandular echogenicity is altered and there is loss of homogeneity due to the presence of multiple hypoechoic or anechoic areas, as well as hyperechoic bands. Loss of definition of the glandular border may also be observed. A single-site substudy of SGUS in TEARS showed that a greater number of patients had improvement in parotid gland echostructure at 24 weeks after rituximab compared with placebo.^15^ Echostructure was assessed on a 0–4 scale that graded the presence of hypoechoic areas as well as hyperechoic bands. SGUS is, however, an operator-dependent technique, and its utility in a multicentre study is uncertain. Here we report the results of a multiobserver, multicentre SGUS substudy of TRACTISS over a longer therapeutic timeframe.

## Methods

The TRACTISS study has been previously described.^6^ Briefly, 133 patients with PSS were randomised 1:1 to 1000 mg rituximab or placebo given at weeks 0, 2, 24 and 26. Patients and clinicians were blind to the randomised allocation. The primary outcome (30% reduction in either oral dryness or fatigue VAS) was assessed at week 48. Methylprednisolone 100 mg was given prior to each infusion of rituximab or placebo. Subjects could consent to an optional SGUS substudy, with assessments at baseline and weeks 16 and 48. The prespecified substudy primary outcome was total ultrasound score (TUS, range 0–11; table 1). Normal salivary gland echogenicity was defined through similarity with the thyroid. The consistency domain scored the extent of heterogeneity introduced by the presence of hypoechoic areas. The definition domain addressed whether the posterior glandular border was normally visible or else incompletely defined or not possible to define. The hypoechoic foci size domain categorised the size of the glandular hypoechoic lesions that were most typical for that patient. Imaging followed a standard sequence including both transverse and longitudinal views of both parotid and submandibular glands, with data recorded by the sonographer on a study proforma. Additional information was collected for each of the four major salivary glands on vascularity of the gland parenchyma assessed by power Doppler, gland echogenicity (normal, heterogenous or hypoechoic), gland margins (well or ill-defined), approximate hypoechoic foci number (0, 1–5, 5–9 and >10), hypoechoic foci size (<3, 3–7 and >8 mm), as well as domains capturing lymph node abnormalities.

**Table 1:** Domains of the total ultrasound score

Domain	Description	Score
Echogenicity	Normal	0
	Hypoechoic	1
Consistency	Normal	0
	Mild heterogeneity	1
	Evident honeycombed	2
	Gross multifocal	3
Definition	Normal	0
	Moderately defined	1
	Ill-defined	2
Glands involved	None	0
	Parotids or submandibular glands	1
	All glands	2
Hypoechoic foci size	None	0
	Small 2–5 mm	1
	Large 5–8 mm non-vascular	2
	Over 8 mm ± vascular	3
Total		0–11

ESSPRI score was calculated as the mean of 0–10 scales for dryness, fatigue and limb pain. The ESSDAI score was scored by the local investigator. Unstimulated whole salivary flow was collected over 15 min, and stimulated whole salivary flow over 10 min following application of citric acid with a cotton swab to the lateral borders of the tongue every 60 s.

TUS was modelled using mixed effects linear regression, including baseline score, patient age, disease duration and time point. Odds of domain improvement were modelled by repeated-measures logistic regression, including baseline score, age, disease duration and time point. Descriptive summary statistics, scatterplots and boxplots were produced to explore and summarise the data.

## Results

In total, 66 patients (49.6%) from the total study population consented to SGUS, and 52 (39.1%; n=26 rituximab and n=26 placebo) patients from nine centres completed the baseline and at least one follow-up visit. Of these 52 patients, 43 (83%) completed all three visits. There were no apparent differences in relevant characteristics between those consenting and not consenting to the substudy (online supplementary table S1). The two arms of the substudy were also similar (table 2), although TUS in the rituximab arm was on average one point greater.

10.1136/annrheumdis-2017-212268.supp1 Supplementary file 1

**Figure 1:**
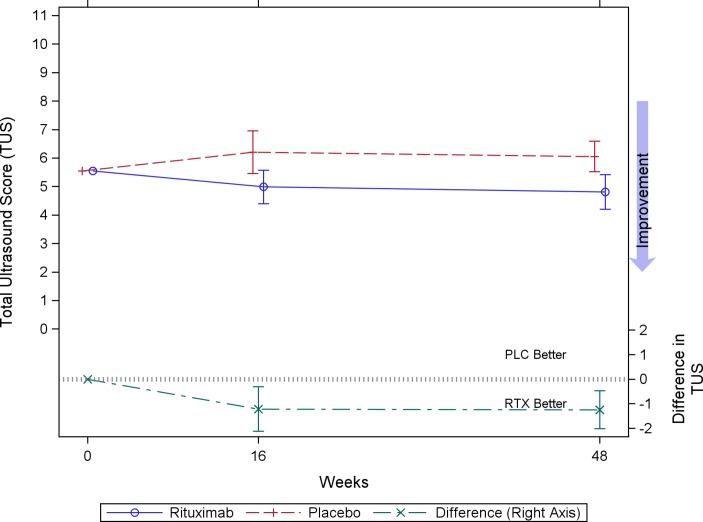
Baseline-adjusted total ultrasound score (TUS) at follow-up. Mean baseline-adjusted TUS, and between-group differences at weeks 16 and 48. Data modelled using a covariance pattern mixed model, with the baseline value fitted as a fixed effect. Values presented are least-squares means and 95% CIs for the two groups, and the differences between the groups. PLC, placebo; RTX, rituximab.

Figure 1 illustrates the baseline-adjusted values of TUS over time, modelling the change from baseline at each time point. Estimated baseline-adjusted TUS at week 16 was 6.2 (95% CI 5.4 to 7.0) for placebo and 5.0 (95% CI 4.4 to 5.6) for rituximab, and at week 48, 6.1 (95% CI 5.5 to 6.6) and 4.8 (95% CI 4.2 to 5.4), respectively. Estimated between-group differences (rituximab-placebo) in baseline-adjusted TUS were −1.2 (95% CI −2.1 to −0.3; P=0.0099) and −1.2 (95% CI −2.0 to –0.5; P=0.0023) at weeks 16 and 48, respectively.

**Table 2:** Selected baseline characteristics of subjects with both baseline and follow-up data in salivary gland ultrasound substudy

	Placebo (n=26)	Rituximab (n=26)	All (n=52)
Age (years)	57.4 (11.1)	56.7 (10.92)	57.1 (10.91)
Years since diagnosis	6.6 (5.67)	5.38 (4.82)	6.0 (5.25)
≥10 years since diagnosis, n (%)	6 (23.1)	4 (15.4)	10 (19.2)
Female sex, n (%)	23 (88.5)	25 (96.2)	48 (92.3)
Current medications (prior to randomisation)			
Pilocarpine, n (%)	1 (3.8)	4 (15.4)	5 (9.6)
Hydroxychloroquine, n (%)	13 (50.0)	15 (57.7)	28 (53.8)
Corticosteroids, n (%)	6 (23.1)	2 (7.7)	8 (15.4)
NSAIDS: n (%)	7 (26.9)	5 (19.2)	12 (23.1)
Unstimulated salivary flow (mL/15 min)	1.4 (2.34)	0.8 (0.71)	1.1 (1.72)
Stimulated salivary flow (mL/10 min)	3.8 (4.08)	3.7 (5.51)	3.7 (4.82)
IgG (g/L)	17.2 (7.67)	17.8 (6.02)	17.5 (6.82)
IgA (g/L)	3.7 (2.87)	3.0 (1.0)	3.3 (2.14)
IgM (g/L)	1.2 (0.64)	1.4 (0.65)	1.28 (0.64)
Anti-Ro autoantibody positive, n (%)	26 (100)	25 (96.2)	51 (98.1)
Reduced C4, n (%)	4 (15.4)	4 (15.4)	8 (15.4)
Visual analogue scales (average over last two weeks, mm; 100=severe, except global)			
Fatigue	74.5 (13.46)	67.0 (18.22)	70.8 (16.30)
Oral dryness	75.6 (15.13)	73.8 (13.30)	74.7 (14.14)
Ocular dryness	64.7 (23.25)	65.7 (19.25)	65.2 (21.09)
Overall dryness	73.4 (15.64)	71.3 (13.17)	72.4 (14.36)
Joint pain	56.4 (28.40)	47.2 (27.21)	51.8 (27.93)
Global assessment (100=PSS very active)	73.4 (14.08)	62.2 (18.90)	67.8 (17.45)
ESSPRI (10=maximal symptom severity)	6.7 (1.63)	6.4 (1.64)	6.6 (1.64)
ESSDAI (123=maximal disease activity)	6.8 (3.82)	5.1 (4.55)	6.0 (4.24)
ESSDAI glandular domain, n (%)			
No activity	17 (65.4)	22 (84.6)	39 (75.0)
Low activity	8 (30.8)	3 (11.5)	11 (21.2)
Moderate activity	1 (3.8)	1 (3.8)	2 (3.8)
TUS	5.02 (3.06)	6.5 (2.04)	5.9 (2.65)
TUS domains			
Echogenicity	0.5 (0.51)	0.8 (0.43)	0.7 (0.48)
Consistency	1.3 (1.00)	1.5 (0.91)	1.4 (0.95)
Definition	0.8 (0.83)	1.3 (0.74)	1.0 (0.82)
Glands involved	1.5 (0.81)	1.9 (0.43)	1.7 (0.67)
Hypoechoic foci size	1.0 (0.68)	1.1 (0.48)	1.1 (0.58)

Values are mean and SD unless otherwise stated.

ESSDAI, European League Against Rheumatism Sjögren’s Syndrome Disease Activity Index; ESSPRI, European Sjögren’s Syndrome Patient Reported Index; NSAID, non-steroidal anti-inflammatory drugs; PSS, primary Sjögren’s syndrome; TUS, total ultrasound score.

For each TUS domain, we fitted a repeated-measures logistic regression to model the odds of a response in the rituximab arm (defined as ≥1-point improvement) as a function of the baseline score, age category, disease duration and time point. Glandular definition was the only domain to show statistically significant improvement with an OR of 6.8 (95% CI 1.1 to 43.0; P=0.043) at week 16 and 10.3 (95% CI 1.0 to 105.9; P=0.050) at week 48 (online supplementary table S2). No difference between rituximab and placebo was observed in any of the additional exploratory ultrasound parameters collected, with the exception of gland margin scores which showed deterioration in the placebo group (mean sum of scores over all glands increasing from 1.8 (SD 1.95) at baseline to 2.4 (SD 1.89) at 48 weeks compared with 2.3 (SD 1.83) to 2.4 (SD 1.97) in the rituximab group).

Improvement of ≥1 point in TUS, compared with no improvement or worsening, was not associated with improvement in unstimulated or stimulated salivary flow rates, ESSPRI score or dryness domain VAS at weeks 16 or 48, in the whole population or when analysing the rituximab arm alone. No associations were observed with ≥1-point improvement in either the glandular definition or hypoechoic foci size domains. TUS did not correlate with total ESSDAI score, the ESSDAI glandular domain or salivary flow rates at any time point, either in the whole population or the rituximab arm. Baseline TUS was not correlated with improvement in salivary flow rates, ESSPRI or oral dryness VAS at either week 16 or 48 in the rituximab arm (data not shown).

## Discussion

We demonstrated a statistically significant improvement in TUS after rituximab compared with placebo. While this observation is similar to that in the TEARS substudy, there are a number of key differences. First, in TRACTISS rituximab was given at baseline and then again at 6 months, with a longer follow-up to 48 weeks. Second, the TRACTISS substudy was larger, multicentre and multiobserver. The ability of ultrasound to detect changes in this setting is important in encouraging further development of this tool. Third, TRACTISS used a composite SGUS score. Fourth and related to the last point, the number and size of hypoechogenic foci showed no change in TRACTISS, in contrast to the TEARS study.

The pathological correlate of the hypoechoic areas observed on ultrasound in PSS is uncertain. In TEARS, there was a correlation between histological focus score and SGUS score, suggesting that hypoechoic areas represent areas of inflammatory cell infiltrate.^16^ Furthermore, both high baseline SGUS score and high numbers of infiltrating B cells were predictive of non-response.^17 18^ However, opposite findings on B cell infiltration and rituximab responsiveness have been reported by Delli *et al*,^19^ and in a cohort of patients with suspected PSS there was only a modest agreement between the same SGUS score and biopsy.^13^ Therefore, it remains possible that the highest grades of hypoechoic lesions might reflect damage as well as inflammation in a subset of patients, explaining why we observed no change in their size or number.

Our results suggest that glandular definition was an important domain driving change in TUS. While there is a pragmatic attractiveness in simplified scores focusing on hypoechoic areas for diagnosis,^20^ our data encourage the collection of a wider range of features/domains in clinical trials as there is yet much to learn about the responsiveness of US to effective treatments in PSS.

The clinical significance of our findings is uncertain. TRACTISS did not meet its primary endpoint,^6^ and no association between TUS improvement and salivary flow was found. We also found no apparent inverse association between salivary flow rates and TUS at baseline, in contrast to previous cross-sectional studies, which may reflect our small sample size given that previously reported correlations were only fair to moderate.^21 22^ Furthermore, the improvement in the glandular definition domain was only of marginal statistical significance. We used a novel composite score, designed to be comprehensive but also pragmatic, but which predated the EULAR pSS working group reference atlas.^23^ Other limitations include the small number of subjects and the multiplicity of statistical comparisons, for which we did not adjust our nominal significance levels. Although the sonographers in this study were experienced in SGUS, ultrasound machines were not standardised between centres, and some domains, especially the definition domain, can be difficult to assess. Intraobserver and interobserver reliability was not studied and could have impacted our findings; further standardisation of SGUS in PSS is urgently required. Arguably, however, the ability to distinguish treatment arms despite such standardisation may increase the relevance of our findings.

There is good reason to believe that rituximab monotherapy may stimulate new autoimmune B cells through elevation in BLyS levels^24^ and may be inefficient at depleting tissue B cells.^25^ The fact that we observed a difference in TUS between study arms despite these limitations encourages further research on B cell depletion therapy in PSS, including use of combination therapies,^26^ and on SGUS as an imaging biomarker.

## References

[1] Cornec D, Devauchelle-Pensec V, Tobón GJ, et al B cells in Sjögren’s syndrome: from pathophysiology to diagnosis and treatment. J Autoimmun 2012;39:161–7. 10.1016/j.jaut.2012.05.014 22749831

[2] Dass S, Bowman SJ, Vital EM, et al Reduction of fatigue in Sjögren syndrome with rituximab: results of a randomised, double-blind, placebo-controlled pilot study. Ann Rheum Dis 2008;67:1541–4. 10.1136/ard.2007.083865 18276741

[3] Meijer JM, Meiners PM, Vissink A, et al Effectiveness of rituximab treatment in primary Sjögren’s syndrome: a randomized, double-blind, placebo-controlled trial. Arthritis Rheum 2010;62:960–8. 10.1002/art.27314 20131246

[4] Ciccia F, Guggino G, Rizzo A, et al Rituximab modulates IL-17 expression in the salivary glands of patients with primary Sjögren’s syndrome. Rheumatology 2014;53:1313–20. 10.1093/rheumatology/keu004 24602921

[5] Palanichamy A, Jahn S, Nickles D, et al Rituximab efficiently depletes increased CD20-expressing T cells in multiple sclerosis patients. J Immunol 2014;193:580–6. 10.4049/jimmunol.1400118 24928997 PMC4082756

[6] Bowman SJ, Everett CC, O’Dwyer JL, et al Randomized controlled trial of rituximab and cost-effectiveness analysis in treating fatigue and oral dryness in primary sjögren’s syndrome. Arthritis Rheumatol 2017;69:1440–50. 10.1002/art.40093 28296257

[7] Devauchelle-Pensec V, Mariette X, Jousse-Joulin S, et al Treatment of primary Sjögren syndrome with rituximab: a randomized trial. Ann Intern Med 2014;160:233–42. 10.7326/M13-1085 24727841

[8] Seror R, Theander E, Bootsma H, et al Outcome measures for primary Sjögren’s syndrome: a comprehensive review. J Autoimmun 2014;51:51–6. 10.1016/j.jaut.2013.12.010 24411404

[9] Jorkjend L, Johansson A, Johansson AK, et al Resting and stimulated whole salivary flow rates in Sjögren’s syndrome patients over time: a diagnostic aid for subsidized dental care? Acta Odontol Scand 2004;62:264–8. 10.1080/00016350410001702 15841813

[10] Fisher BA, Brown RM, Bowman SJ, et al A review of salivary gland histopathology in primary Sjögren’s syndrome with a focus on its potential as a clinical trials biomarker. Ann Rheum Dis 2015;74:1645–50. 10.1136/annrheumdis-2015-207499 26034044

[11] Fisher BA, Jonsson R, Daniels T, et al Standardisation of labial salivary gland histopathology in clinical trials in primary sjögren’s syndrome. Ann Rheum Dis 2017;76:1161–8. 10.1136/annrheumdis-2016-210448 27965259 PMC5530351

[12] Baldini C, Luciano N, Mosca M, et al Salivary gland ultrasonography in sjögren’s syndrome: clinical usefulness and future perspectives. Isr Med Assoc J 2016;18:193–6. 27228642

[13] Cornec D, Jousse-Joulin S, Pers JO, et al Contribution of salivary gland ultrasonography to the diagnosis of Sjögren’s syndrome: toward new diagnostic criteria? Arthritis Rheum 2013;65:216–25. 10.1002/art.37698 23108632

[14] Jousse-Joulin S, Milic V, Jonsson MV, et al Is salivary gland ultrasonography a useful tool in Sjögren’s syndrome? A systematic review. Rheumatology 2016;55:789–800. 10.1093/rheumatology/kev385 26667216

[15] Jousse-Joulin S, Devauchelle-Pensec V, Cornec D, et al Brief report: ultrasonographic assessment of salivary gland response to rituximab in primary sjögren’s syndrome. Arthritis Rheumatol 2015;67:1623–8. 10.1002/art.39088 25708147

[16] Cornec D, Jousse-Joulin S, Costa S, et al High-grade salivary-gland involvement, assessed by histology or ultrasonography, is associated with a poor response to a single rituximab course in primary sjögren’s syndrome: data from the TEARS randomized trial. PLoS One 2016;11:e0162787 10.1371/journal.pone.0162787 27662653 PMC5035078

[17] Cornec D, Costa S, Devauchelle-Pensec V, et al Do high numbers of salivary gland-infiltrating B cells predict better or worse outcomes after rituximab in patients with primary Sjögren’s syndrome? Ann Rheum Dis 2016;75:e33 10.1136/annrheumdis-2016-209300 26895746

[18] Cornec D, Costa S, Devauchelle-Pensec V, et al Blood and salivary-gland BAFF-driven B-cell hyperactivity is associated to rituximab inefficacy in primary Sjögren’s syndrome. J Autoimmun 2016;67:102–10. 10.1016/j.jaut.2015.11.002 26688003

[19] Delli K, Haacke EA, Kroese FG, et al Towards personalised treatment in primary Sjögren’s syndrome: baseline parotid histopathology predicts responsiveness to rituximab treatment. Ann Rheum Dis 2016;75:1933–8. 10.1136/annrheumdis-2015-208304 26757748

[20] Theander E, Mandl T Primary Sjögren’s syndrome: diagnostic and prognostic value of salivary gland ultrasonography using a simplified scoring system. Arthritis Care Res 2014;66:1102–7. 10.1002/acr.22264 24339361

[21] Baldini C, Luciano N, Tarantini G, et al Salivary gland ultrasonography: a highly specific tool for the early diagnosis of primary Sjögren’s syndrome. Arthritis Res Ther 2015;17:146 10.1186/s13075-015-0657-7 26022533 PMC4461980

[22] Mossel E, Delli K, van Nimwegen JF, et al Ultrasonography of major salivary glands compared with parotid and labial gland biopsy and classification criteria in patients with clinically suspected primary Sjögren’s syndrome. Ann Rheum Dis 2017;76:1883–9. 10.1136/annrheumdis-2017-211250 28754802

[23] Jousse-Joulin S, Nowak E, Cornec D, et al Salivary gland ultrasound abnormalities in primary Sjögren’s syndrome: consensual US-SG core items definition and reliability. RMD Open 2017;3:e000364 10.1136/rmdopen-2016-000364 28879042 PMC5575597

[24] Cambridge G, Stohl W, Leandro MJ, et al Circulating levels of B lymphocyte stimulator in patients with rheumatoid arthritis following rituximab treatment: relationships with B cell depletion, circulating antibodies, and clinical relapse. Arthritis Rheum 2006;54:723–32. 10.1002/art.21650 16508933

[25] Lin W, Seshasayee D, Lee WP, et al Dual B cell immunotherapy is superior to individual anti-CD20 depletion or BAFF blockade in murine models of spontaneous or accelerated lupus. Arthritis Rheumatol 2015;67:215–24. 10.1002/art.38907 25303150 PMC4312898

[26] De Vita S, Quartuccio L, Salvin S, et al Sequential therapy with belimumab followed by rituximab in Sjögren’s syndrome associated with B-cell lymphoproliferation and overexpression of BAFF: evidence for long-term efficacy. Clin Exp Rheumatol 2014;32:490–4. 24802131

[27] Fisher B, Everett C, Rout J, et al Effect of rituximab on a salivary gland ultrasound score in primary sjögren’s syndrome: results of multicentre double-blind randomised controlled trial sub-study (abstract). Arthritis Rheumatol 2017;69.

