# Supplementary material for: Effect of rituximab on a salivary gland ultrasound score in primary Sjögren’s syndrome: results of the TRACTISS randomised double-blind multicentre substudy
Source: Ann Rheum Dis. 2017 Dec 23;77(3):412–6. doi: 10.1136/annrheumdis-2017-212268 (PMC5867400; doi:10.1136/annrheumdis-2017-212268)
Supplement: Supplementary file 1 [file annrheumdis-2017-212268supp001.docx]

**Effect of rituximab on a salivary gland ultrasound score in primary Sjögren’s syndrome: results of the TRACTISS randomised double-blind multicentre sub-study**

*Benjamin A. Fisher^1,2,3^, Colin C Everett ^4^, John Rout^5^, John L O’Dwyer ^6^, Paul Emery ^7^, Costantino Pitzalis ^8^, Wan-fai Ng ^9^, Andrew Carr^10^, Colin T Pease ^7^, Elizabeth J Price ^11^, Nurhan Sutcliffe ^12^, Jimmy Makdissi^13^, Anwar R Tappuni^13^, Nagui ST Gendi^14^, Frances C Hall ^15^, Sharon P Ruddock ^4^, Catherine Fernandez ^4^, Claire T Hulme ^6^, Kevin A Davies ^16^, Christopher J Edwards ^17^, Peter C Lanyon ^18^, Robert J Moots^19^, Euthalia Roussou^20^, Andrea Richards^5^, Linda D Sharples^21^, Michele Bombardieri ^8^ and Simon J Bowman ^1,2,3^*

Online Supplementary Table S1. Selected baseline characteristics of subjects consenting and not-consenting to the salivary gland ultrasound (SGUS) substudy.

|  | | Did not consent (n=67) | Consented to SGUS substudy (n=66) | All (n=133) |
| --- | --- | --- | --- | --- |
| Age (Years) | | 52.8 (11.24) | 56.0 (11.66) | 54.4 (11.5) |
| Years since diagnosis | | 6.1 (5.82) | 5.4 (4.92) | 5.7 (5.4) |
| 10 or more years since diagnosis: n (%) | | 14 (20.9) | 10 (15.2) | 24 (18.0) |
| Female Sex: n (%) | | 62 (92.5) | 62 (93.9) | 124 (93.2) |
| Current Medications (prior to randomisation) | |  |  |  |
|  | Pilocarpine: n (%) | 5 (7.6) | 9 (13.4) | 14 (10.5) |
|  | Hydroxychloroquine: n (%) | 34 (51.5) | 40 (59.7) | 74 (55.6) |
|  | Corticosteroids: n (%) | 8 (12.1) | 11 (16.4) | 19 (14.3) |
|  | NSAIDS: n (%) | 15 (22.7) | 20 (29.9) | 35 (26.4) |
| Unstimulated Salivary Flow (mL/15min) | | 1.1 (1.25) | 1.2 (1.77) | 1.2 (1.52) |
| Stimulated salivary flow (mL/10min) | | 3.4 (4.10) | 3.5 (4.43) | 3.4 (4.26) |
| IgG (g/L) | | 18.8 (8.19) | 17.2 (6.8) | 18.0 (7.5) |
| IgA (g/L) | | 3.2 (1.33) | 3.2 (1.98) | 3.2 (1.7) |
| IgM (g/L) | | 1.1 (0.52) | 1.3 (0.71) | 1.2 (0.6) |
| Anti-Ro autoantibody positive | | 67 (100.0) | 65 (98.5) | 132 (99.2) |
| Reduced C4 | | 9 (13.4) | 10 (15.2) | 19 (14.3) |
| Visual Analogue Scales (Average over last 2 weeks, mm. 100=Severe, except Global) | |  |  |  |
|  | Fatigue | 71.7 (16.37) | 74.0 (15.87) | 72.8 (16.1) |
|  | Oral Dryness | 74.0 (14.61) | 78.6 (17.43) | 76.3 (16.2) |
|  | Ocular Dryness | 65.0 (20.20) | 76.4 (18.68) | 70.7 (20.2) |
|  | Overall Dryness | 72.2 (14.07) | 78.3 (17.00) | 75.2 (15.8) |
|  | Joint Pain | 50.7 (28.32) | 58.8 (27.26) | 54.7 (28.0) |
|  | Global Assessment (100=PSS very active) | 68.1 (17.27) | 71.2 (18.47) | 69.7 (17.9) |
| ESSPRI (10=Maximal Symptom Severity) | | 6.7 (1.71) | 6.5 (1.57) | 6.6 (1.6) |
| ESSDAI (123=Maximal Disease Activity) | | 5.6 (5.10) | 5.7 (3.91) | 5.7 (4.52) |

Footnote: Values are Mean and standard deviation unless otherwise stated

Online Supplementary Table S2. Odds of rituximab improving the individual domains of the Total Ultrasound Score were modelled by repeated measures logistic regression, including baseline score, age, disease duration and time-point.

| Domain | Week 16 | Week 48 |
| --- | --- | --- |
| Echogenicity | 0.20  (0.02, 2.8)  *p=0.23* | 1.89  (0.21, 17.34  *p=0.57* |
| Consistency | 5.09  (0.85, 30.50)  *p=0.07* | 4.88  (0.69, 34.33)  *p=0.11* |
| Definition | 6.76  (1.06, 42.98)  ***p=0.04*** | 10.31  (1.00, 105.89)  ***p=0.05*** |
| Glands involved | - | 4.98  (0.47, 53.02)  *p=0.18* |
| Hypoechoic foci size | 0.46  (0.05, 4.64)  *p=0.51* | 1.80  (0.23, 13.98)  *p=0.57* |

Footnote: Values are odds ratios (95% confidence interval)
